# Le carcinome tubulo-mucineux et fusiforme: une tumeur rénale rare

**DOI:** 10.11604/pamj.2017.26.187.10456

**Published:** 2017-03-30

**Authors:** Oussama Ziouani, Abdelilah Elalaoui, Hicham Elbote, Salwa Belhabib, Hachem El Sayegh, Ali Iken, Lounis Benslimane, Fouad Zouaidia, Yassine Nouini

**Affiliations:** 1 Service d’Urologie A, Hôpital Ibn Sina, CHU Rabat, Maroc; 2 Service d’Anatomie Pathologique, Hôpital Ibn Sina, CHU Rabat, Maroc

**Keywords:** Rein, carcinome rénal, carcinome tubulomucineux et fusiforme, bas grade, Kidney, renal carcinoma, mucinous tubular and spindle cell carcinoma, low grade

## Abstract

Le carcinome tubulo-mucineux et fusiforme désigne une tumeur rare décrite dans la classification OMS 2004 comme une nouvelle entité. Elle est reconnue de comportement relativement indolent. Nous rapportons l'observation d'une femme âgée de 60 ans qui présentait des douleurs lombaires, et chez qui la tomodensitométrie a révélé la présence d'une masse rénale gauche mesurant 55 x 40 mm. La patiente a été traitée par néphrectomie partielle gauche dont l'examen macroscopique a montré la présence d'une tumeur bien limitée d'aspect charnu avec des remaniements hémorragiques et nécrotiques. L'étude histologique a confirmé le carcinome tubulo-mucineux et à cellules fusiformes de bas grade. L'immunohistochimie a révélé une positivité à la cytokératine (CK 7 et CK 19) et à l'antigène des membranes épithéliales (EMA), et une négativité au CD10. L'évolution était favorable avec un recul de 6 mois.

Mucinous tubular and spindle cell carcinoma is a rare tumor defined in the 2004 WHO classification as a new entity. It is characterized by a relatively indolent behavior We report the case of a 60-year old woman presenting with lumbar pain. CT scan showed left renal mass measuring 55 x 40 mm. The patient underwent left partial nephrectomy. Macroscopic examination showed a well limited fleshy tumor with hemorrhagic or necrotic elements. Histological examination confirmed low grade mucinous tubular and spindle cell carcinoma. Immunohistochemistry revealed cytokeratin positive cells (CK 7 and CK 19), epithelial membrane antigen positive cells (EMA), and CD10-negativity cells. Patient's evolution was favorable, with a follow-up period of 6 months.

## Introduction

Le carcinome tubulomucineux et fusiforme du rein (CTMF) est une entité rare, initialement décrite en 1998 [1]. Elle a été introduite dans la dernière classification de l'OMS 2004 des tumeurs rénales comme une entité distincte [2]. Nous rapportons le cas d'un carcinome tubulomucineux et fusiforme de faible grade chez une femme de 60 ans. L'objectif de notre travail est de rapporter un cas de cette entité rare dont la connaissance par les cliniciens et les pathologistes est primordiale, du fait de son pronostic favorable comparé à celui des autres carcinomes à cellules rénales.

## Patient et observation

**Figure 1:**
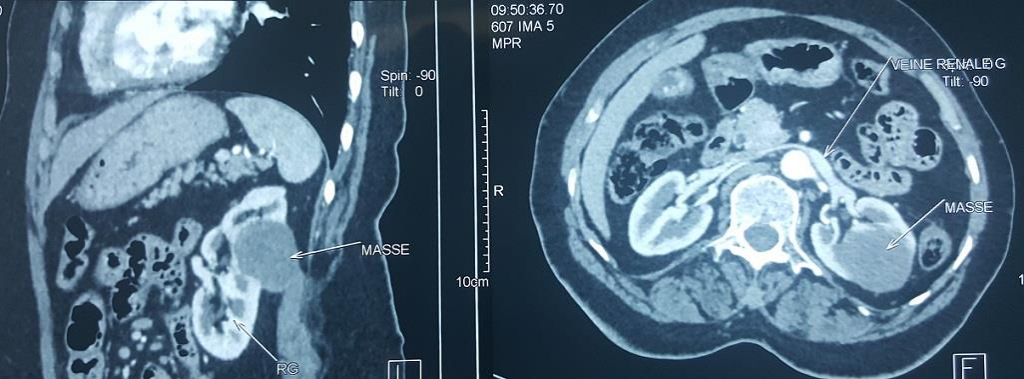
Examen tomodensitométrique montrant une masse médio-rénale gauche

**Figure 2:**
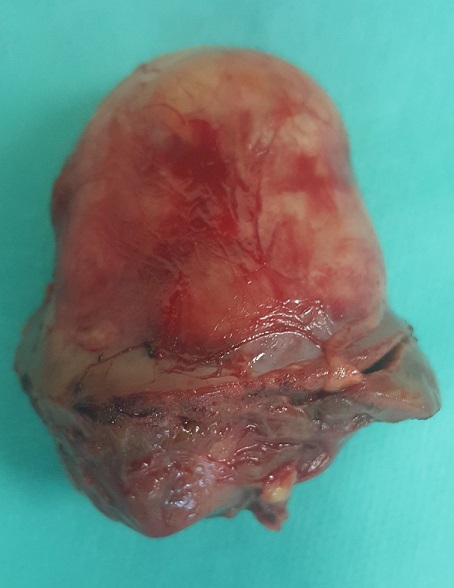
Pièce de néphrectomie partielle gauche comportant la tumeur

**Figure 3:**
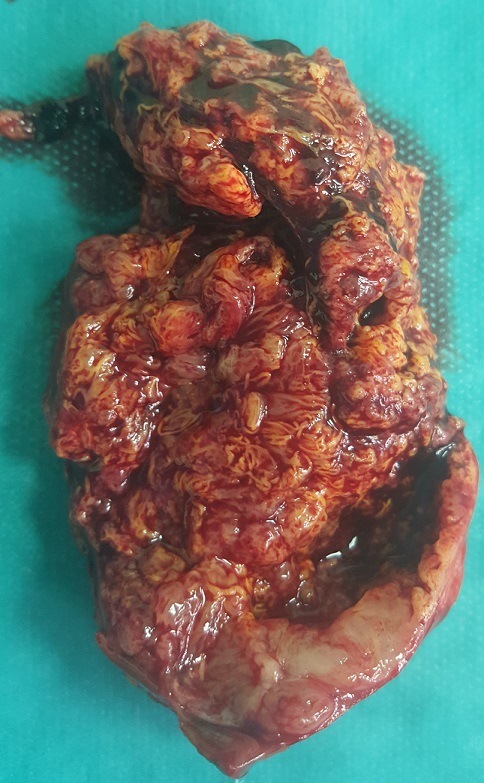
Aspect charnu de la tumeur à la coupe avec des zones hémorragiques et nécrotiques

**Figure 4:**
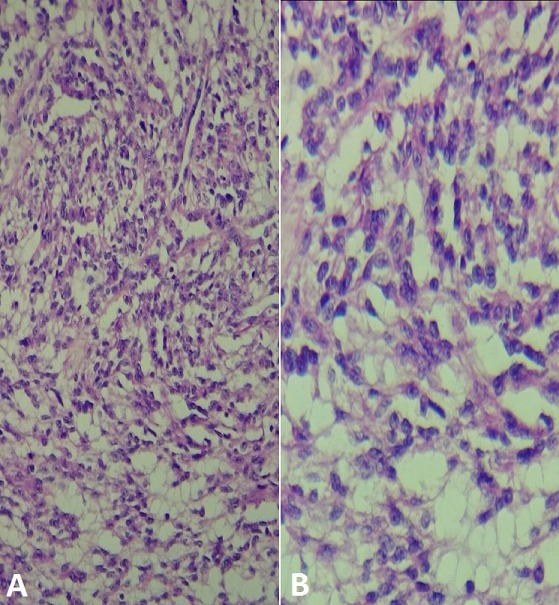
Aspect histologique montrant une prolifération carcinomateuse tubulaire et fusiforme au sein d’un stroma myxoïde (A: x250, B: x400)

Une femme âgée de 60 ans, sans antécédent pathologique particulier, a été hospitalisée pour la prise en charge de douleurs lombaires gauches sans notion d'hématurie macroscopique évoluant depuis 6 mois. L'examen clinique et le bilan biologique étaient sans particularité. La tomodensitométrie (TDM) a révélé la présence d'une masse médio rénale gauche bien limitée mesurant 55x40 mm sans calcification ni nécrose (Figure 1). Le bilan d'extension locorégional était négatif. La patiente a bénéficié d'une néphrectomie partielle gauche permettant l'exérèse d'une tumeur solide, bien limitée mesurant 5x3 cm (Figure 2). Elle était charnue à la coupe avec des zones de remaniements hémorragiques et nécrotiques (Figure 3). Histologiquement, il s'agit d'une prolifération tumorale carcinomateuse faite de tubules allongés bordés par des cellules à inflexion fusiforme (Figure 4). Ces tubules sont séparés par un stroma mucineux. Les cellules tumorales isolées sont de petites tailles, cubiques ou ovales, avec peu d'atypies cytonucléaires. L'analyse immunohistochimique a révélé une positivité des cellules tumorales à la CK7, CK19 et à l'EMA. L'ensemble des constatations macroscopiques, histologiques et immunohistochimiques a conclu à un carcinome tubulomucineux et fusiforme du rein. Les suites opératoires de la patiente étaient simples, et le suivi postopératoire pendant 6 mois était sans récidive ni métastase.

## Discussion

Depuis sa première description en 1998, plusieurs cas isolés ou petites séries de carcinome tubulomucineux et fusiforme ont été rapportés [3]. Cette tumeur est observée chez l'adulte d'âge moyen (5ème décénnie) avec une nette prédilection féminine (sex-ratio 1/3) [3]. Il s'agit d'un type rare de carcinome à cellules rénales composé de néoplasmes épithéliaux de bas grade avec des caractéristiques tubulomucineuses et des cellules fusiformes [4]. Sur le plan clinique, occasionnellement, lorsque les lésions sont grandes, les patients peuvent présenter des douleurs au flanc ou une hématurie. La majorité de ces tumeurs sont accidentellement découvertes lors d'examens par imagerie abdominale suite à d'autres indications. [5]. Leur taille est variable allant de moins de 1 cm à plus de 18 cm de diamètre. Les caractéristiques radiologiques peuvent ressembler à d'autres variantes de carcinome à cellules rénales, tel le carcinome chromophobe ou papillaire, qui ont un pronostic moins favorable [5].

Cependant, le CTMF devrait être soupçonné devant une lésion de taille importante, circonscrite, faiblement rehaussée avec un signal hypo-intense à intermédiaire sur une image en T2 pondérée [5]. Macroscopiquement, ces tumeurs sont essentiellement bien limitées, fermes, souvent homogènes, d'aspect gris-blanchâtre ou plus rarement brunâtre. Les remaniements hémorragiques ou nécrotiques sont rares [6]. Le CTMF se caractérise par son pronostic favorable, sa localisation médullaire et sa morphologie particulière associant une architecture tubulaire et fusiforme au sein d'un stroma distinctement myxoïde [3, 4]. Les contingents fusiforme et tubulaire sont d'abondance variable selon les cas, mais montrent toujours un faible grade nucléaire [3]. Ces tumeurs présentent un profil immunohistochimique complexe, exprimant aussi bien des marqueurs du néphron distal (EMA, CK19, CK7, E-cadhérine) que des marqueurs du tube proximal (RCC Ma, AMACR et CD15) [3]. Dans la littérature, les données cytogénétiques montrent diverses anomalies portant sur un nombre variables de chromosomes, mais il n'a jamais été observé une perte du chromosome 3p caractéristique des carcinomes à cellules claires [7]. Le principal diagnostic différentiel du CTMF est le carcinome papillaire du rein dans sa variante compacte d'autant plus que ces deux tumeurs présentent une similitude histologique et immunohistochimique, ce qui a conduit certains auteurs à considérer le CTMF comme une variante du carcinome papillaire type 1 [3]. Cependant, les études cytogénétiques vont à l'encontre de cette hypothèse puisqu'il n'a jamais été démontré des anomalies spécifiques des carcinomes papillaires (trisomie 7 et 17, perte du chromosome Y) [7, 8]. Enfin la composante à cellules fusiformes du CTMF peut évoquer un carcinome sarcomatoïde mais celui-ci se reconnaît par ses mitoses et son pléomorphisme nucléaire [7]. Le CTMF peut lui-même subir une tranformation sarcomatoïde [9].

Le pronoctic du carcinome tubulomucineux et fusiforme est généralement favorable en accord avec son caractère histologique de bas grade, et l'excision chirurgicale complète semble être le traitement adéquat [2, 10]. Les rares métastases qui ont été rapportées sont habituellement associées à des tumeurs de haut grade ou ayant subi une transformation sarcomatoïde [7, 10]. Sur le plan thérapeutique, les patients avec des tumeurs localisées sont généralement traités par excision chirurgicale complète en l'occurrence une néphrectomie partielle ou totale [10]. Pour les formes métastatiques, un cas montrant une réponse au sunitinib a été documenté [11].

## Conclusion

Il est primordial que les pathologistes et les cliniciens sachent reconnaître le carcinome tubulomucineux et fusiforme du fait de son pronostic favorable. D'autres études moléculaires sont nécessaires pour mieux clarifier l'histogenèse de cette tumeur.
